# Dynamic Boronic Ester Cross-Linked Polymers with Tunable Properties via Side-Group Engineering

**DOI:** 10.3390/polym16243567

**Published:** 2024-12-20

**Authors:** Linlin Wang, Xiao Wang, Shengyu Feng, Lei Li

**Affiliations:** Key Laboratory of Special Functional Aggregated Materials of Ministry of Education, Shandong Key Laboratory of Advanced Silicone Materials and Technology, School of Chemistry and Chemical Engineering, Shandong University, Jinan 250100, China

**Keywords:** poly(β-hydroxyl amine)s, boronic ester, dynamic, healing, reprocessable

## Abstract

The development of dynamic covalent materials with repairability, reprocessability, and recyclability is crucial for sustainable development. In this work, we report a new strategy to adjust the thermomechanical properties of boronic ester cross-linked poly(β-hydroxyl amine)s through side-group engineering. By tuning the side groups of the poly(β-hydroxyl amine)s, we have developed self-healable, reprocessable, and shape-programmable materials. By tuning the side groups of the poly(β-hydroxyl amine)s, the thermomechanical properties can be readily adjusted. Notably, the 3-amino-1,2-propanediol-derived polymer exhibits enhanced thermal (T_g_ = 95 °C) and mechanical (tensile strength = 34.2 MPa) performance due to increased hydrogen bonding. Benefiting from the dynamic reversibility of the boronic esters, these materials demonstrate solvent-assisted healing, reprocessing, chemical recycling, and shape programming capabilities. Given their straightforward synthesis, tunable properties, and robust dynamic features, the boronic ester cross-linked poly(β-hydroxyl amine)s hold great promise for various applications, including flexible electronic and biomedical materials.

## 1. Introduction

The widespread use of thermosets in industry and daily life is due to their outstanding mechanical, thermal, and chemical properties. However, the permanent cross-linking in thermosets makes them unable to be reprocessed or recycled once cured, leading to environmental pollution and resource waste [1,2]. To address this challenge, the development of covalent adaptable networks (CANs) has emerged as a promising sustainable alternative [3]. CANs are polymer networks with reversible covalent cross-links that can be reorganized under external stimuli. This allows CANs to maintain the desirable properties of thermosets while also enabling reprocessing, recycling, self-healing, and other adaptive behaviors [4,5]. The introduction of dynamic covalent bonds in polymer networks provides both mechanical strength and the plasticity required for a circular economy. Many dynamic covalent bond exchange mechanisms have been developed, which respond to various external stimuli such as light, temperature, and pH. These include ester exchange reactions [6], reversible Diels–Alder additions [7], disulfide exchanges, imine exchanges [8], silyl ether exchanges [9,10], and vinylogous urethane exchanges [11].

Boronic ester bonds exhibit unique advantages among dynamic covalent bonds. They can be easily synthesized in a catalyst- and solvent-free manner by the condensation of boronic acids and compounds with hydroxyl groups [12,13]. Moreover, boronic ester bonds can undergo reversible cleavage and reformation in response to stimuli such as temperature, pH, and guest molecules, enabling dynamic and tunable properties [4]. Boronic ester polymers have been systematically investigated in the preparation of self-healing or reprocessable polymeric materials [14,15,16]. Further incorporation of nitrogen (N) atoms as donors to coordinate with boron (B) atoms can effectively improve the thermal stability and transesterification rate of boronic ester cross-linked materials [17]. The N → B coordination, similar in bond energy to hydrogen bonding and metal–ligand interactions, can accelerate the reshuffling of dioxaborolane linkages, leading to enhanced self-healing and toughness [18].

Poly(β-hydroxyl amine)s can be synthesized by the polymerization of amino–epoxy compounds, which can occur at room temperature without the need for a catalyst [19]. By using the diol formed from epoxide ring-opening as the reactive unit and adding boronic acid, dynamic boronic ester cross-linking points can be introduced [20,21,22,23]. The convenience of polymerization and cross-linking of poly(β-hydroxyl amine)s makes it a great choice for dynamic polymer materials. For example, Wu and co-workers developed highly stretchable CANs via the one-pot reaction of poly(propylene glycol) diglycidyl ether, 3-amino-1,2-propanediol, and benzene-1,4-diboronic acid, whose linear viscoelasticity followed the time–temperature superposition principle [24]. Xie and co-workers synthesized a new boronic-ester-containing diamine cross-linker to prepare a reprocessable and ultra-tough epoxy thermoset [25]. Given the structural diversity of poly(β-hydroxyl amine)s, it is necessary to further develop new, versatile, and tunable boronic ester cross-linked poly(β-hydroxyl amine) materials.

Here, we propose a new strategy to adjust the thermomechanical properties of boronic ester cross-linked poly(β-hydroxyl amine)s by side-group engineering and have developed self-healable, reprocessable, and shape-programmable materials. Three different poly(β-hydroxyl amine)s were synthesized by the amine–epoxy click reaction between 1,3-bis(3-glycidyloxypropyl)tetramethyldisiloxane and different primary amines (*n*-butylamine, ethanolamine, and 3-amino-1,2-propanediol). The diethanolamine structure on the polymer chains can form a B-N coordinated boronic ester cross-linking network with benzene-1,4-diboronic acid. The material properties show a strong dependence on the side groups, with the 3-amino-1,2-propanediol-derived polymer exhibiting enhanced thermal and mechanical performance due to the increased hydrogen bonding from the additional hydroxyl groups. Benefiting from the dynamic nature of the boronic esters, the materials demonstrate solvent-assisted healing, reprocessability, and chemical recyclability, making them a promising candidate for circular thermoset materials. Moreover, these materials possess shape-programming capabilities, allowing 2D planar samples to be transformed into various 2D or 3D shapes under mild conditions.

## 2. Materials and Methods

### 2.1. Materials

1,3-Bis(3-glycidyloxypropyl)tetramethyldisiloxane (97%), *n*-butylamine and ethanolamine (99%) were purchased from Beijing HWRK Chem Co., Ltd. (Beijing, China). 3-Amino-1,2-propanediol (98%) and benzene-1,4-diboronic acid (98%) were supplied by Tianjin HEOWNS Biochemical Technology Co., Ltd. (Tianjin, China). All the reagents and solvents were used as received.

### 2.2. Characterization and Measurements

Proton nuclear magnetic resonance (^1^H NMR) spectrum was analyzed on a Bruker AVANCE 400 MHz spectrometer (Bruker Corporation, Ettlingen, Germany)at 25 °C with CDCl_3_ as the solvent and without tetramethylsilane (TMS) as the internal reference for chemical shifts (δ, ppm). Fourier transform infrared spectrum (FT-IR) was recorded on a Bruker TENSOR 27 infrared spectrophotometer with the KBr pellet technique from 4000 cm^−1^ to 500 cm^−1^. The resolution was 4 cm^−1^ and 16 scans. Differential scanning calorimetry (DSC) was measured through SDTQ 600 of TA Instrument (TA Instruments, Newark, USA) under the dry nitrogen flow, and the temperature range was from −140 °C to 100 °C, with a heating and cooling speed of 10 °C/min. For each specimen, two cooling–heating cycles were applied to eliminate thermal history, and the T_g_ was obtained from the second cooling–heating process. The transmittance spectra of the elastomer were also recorded by a Beijing TU-1901 double-beam UV-vis spectrophotometer in the wavelength range of 200–700 nm. Mechanical tensile tests (including the healing and reprocessing tests) of the elastic films were implemented by the Instron 3343 material testing system with a speed of 100 mm/min. For all the tensile tests, the films were cut into several dumbbell-shaped samples with a size of 40 × 4 × 0.8 mm^3^, and at least three parallel tests were conducted.

### 2.3. Synthesis of Polymers PA-1, PA-2, and PA-3 via the Amine-Epoxy Polymerization

The mixture of 1,3-bis(3-glycidyloxypropyl)tetramethyldisiloxane (1 mmol) and *n*-butylamine (1 mmol) was added into methanol (1 mL) and stirred with a mechanical stirrer at 60 °C for 24 h. Subsequently, the crude mixture was diluted with a small amount of THF and slowly precipitated in a large excess of petroleum ether three times. After vacuum drying, the target **PA-1** was obtained.

The mixture of 1,3-bis(3-glycidyloxypropyl)tetramethyldisiloxane (1 mmol) and ethanolamine (1 mmol) was added into methanol (1 mL) and stirred with a mechanical stirrer at 60 °C for 24 h. Subsequently, the crude mixture was diluted with a small amount of THF and slowly precipitated in a large excess of petroleum ether three times. After vacuum drying, the target **PA-2** was obtained.

The mixture of 1,3-bis(3-glycidyloxypropyl)tetramethyldisiloxane (1 mmol) and 3-amino-1,2-propanediol (1 mmol) was added into methanol (1 mL) and stirred with a mechanical stirrer at 60 °C for 24 h. Subsequently, the crude mixture was diluted with a small amount of THF and slowly precipitated in a large excess of petroleum ether three times. After vacuum drying, the target **PA-3** was obtained.

### 2.4. Preparation of Boronic Ester Cross-Linked PB-1, PB-2, and PB-3 [24]

Poly(β-hydroxyl amine) **PA-1** (0.0076 mol of nitrogen atoms) was dissolved in 4 mL of tetrahydrofuran (THF). Separately, benzene-1,4-diboronic acid (0.0076 mol of boron atoms) was dissolved in 8 mL of THF. The boronic acid solution was then added dropwise to the polymer solution, immediately resulting in the formation of a gel. After vacuum drying at 60 °C for 3 days, the obtained material was hot-pressed into tablets at 150 °C and 12 MPa pressure to give sample **PB-1**.

Poly(β-hydroxyl amine) **PA-2** (0.0076 mol of nitrogen atoms) was dissolved in 4 mL of tetrahydrofuran (THF). Separately, benzene-1,4-diboronic acid (0.0076 mol of boron atoms) was dissolved in 8 mL of THF. The boronic acid solution was then added dropwise to the polymer solution, immediately resulting in the formation of a gel. After vacuum drying at 60 °C for 3 days, the obtained material was hot-pressed into tablets at 150 °C and 12 MPa pressure to give sample **PB-2**.

Poly(β-hydroxyl amine) **PA-3** (0.0076 mol of nitrogen atoms) was dissolved in 4 mL of tetrahydrofuran (THF). Separately, benzene-1,4-diboronic acid (0.0076 mol of boron atoms) was dissolved in 8 mL of THF. The boronic acid solution was then added dropwise to the polymer solution, immediately resulting in the formation of a gel. After vacuum drying at 60 °C for 3 days, the obtained material was hot-pressed into tablets at 150 °C and 12 MPa pressure to give sample **PB-3**.

## 3. Results and Discussion

Poly(ß-hydroxyl amine)s were chosen as the basis for constructing dynamic covalent materials due to their abundant hydroxyl groups, which readily form boronic esters with phenylboronic acid. Furthermore, the nitrogen atoms within the polymeric chains establish dative bonds with boron atoms, thereby enhancing the stability of the boronic ester bonds and overall dynamic covalent bond stability. The synthesis of these materials is straightforward via amine–epoxy polymerization, representing an environmentally friendly and highly efficient process that operates without the need for solvents, catalysts, or strictly anhydrous and oxygen-free conditions.

### 3.1. Synthesis of Boronic Ester Cross-Linked Materials

**Scheme 1:**
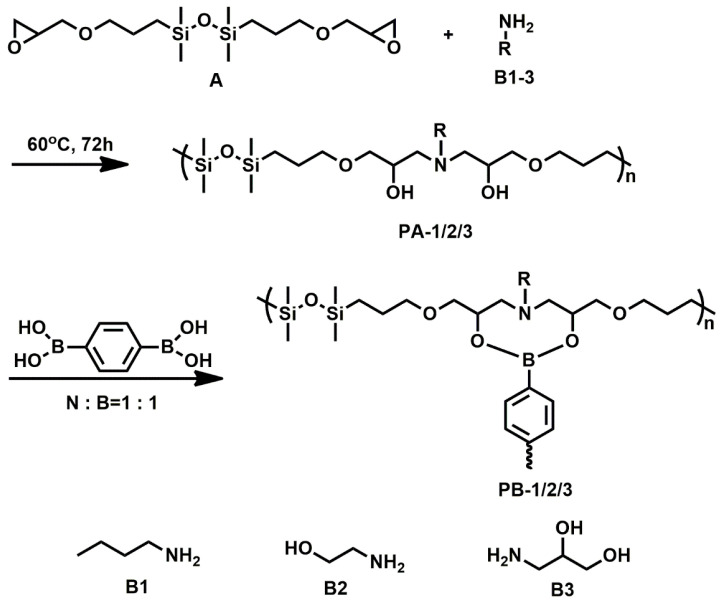
Synthesis of polymers **PA-1**, **PA-2**, and **PA-3** through amine–epoxy polymerization between 1,3-bis(3-glycidyloxypropyl)tetramethyldisiloxane (**A**) and *n*-butylamine (**B1**), ethanolamine (**B2**), and 3-amino-1,2-propanediol (**B3**), and fabrication of their boronic ester cross-linked materials **PB-1**, **PB-2**, and **PB-3**. (For simplicity, the other possible boronic ester isomers that could form have been omitted).

**Figure 1:**
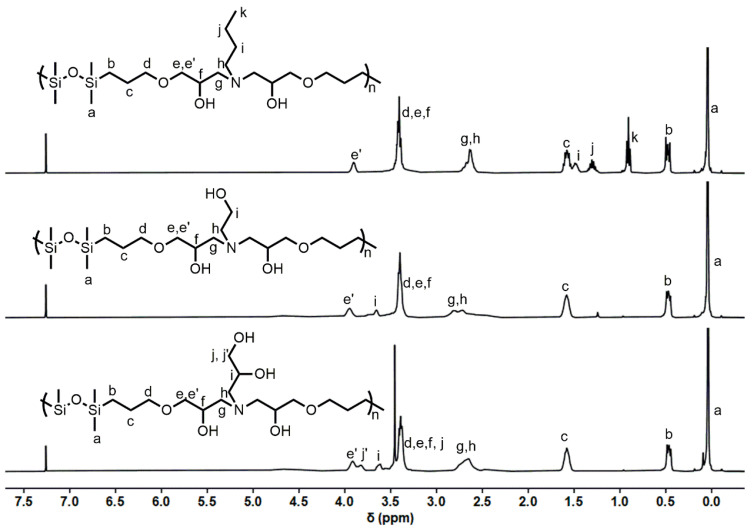
^1^H NMR spectra of polymers **PA-1** (**top**), **PA-2** (**middle**), and **PA-3** (**bottom**). (The different lowercase letters in figure caption represent the proton peak for the corresponding hydrogen atom in polymers).

As shown in Scheme 1, three poly(ß-hydroxyl amine)s were synthesized through amine–epoxy polymerization utilizing commercially available 1,3-bis(3-glycidyloxypropyl)tetramethyldisiloxane (**A**) and different primary amines, namely *n*-butylamine (**B1**), ethanolamine (**B2**), and 3-amino-1,2-propanediol (**B3**). The resulting polymers, designated as polymer **PA-1**, **PA-2**, and **PA-3**, were subjected to characterization using ^1^H NMR spectra. The observed chemical shifts and integral areas, as depicted in Figure 1, were consistent with the theoretical molecular formula, confirming the successful synthesis of the three poly(ß-hydroxyl amine)s.

Subsequently, the introduction of the cross-linking agent benzene-1,4-diboronic acid to the poly(ß-hydroxyl amine)s enabled the fabrication of boronic ester bond cross-linked materials. This led to the preparation of materials **PB-1**, **PB-2**, or **PB-3** from polymers **PA-1**, **PA-2**, or **PA-3**, respectively.

The molar ratio of nitrogen atoms in poly(ß-hydroxyl amine)s to boron atoms in benzene-1,4-diboronic acid was maintained at 1:1. Upon the addition of the solution of benzene-1,4-diboronic acid in THF to the poly(ß-hydroxyl amine) solution in THF, rapid sol-to-gel transition was observed, indicating the high efficiency of the boronic ester crosslinking reaction. Subsequent vacuum drying at 60 °C for 3 days and hot pressing resulted in the formation of the crosslinked materials.

**Figure 2:**
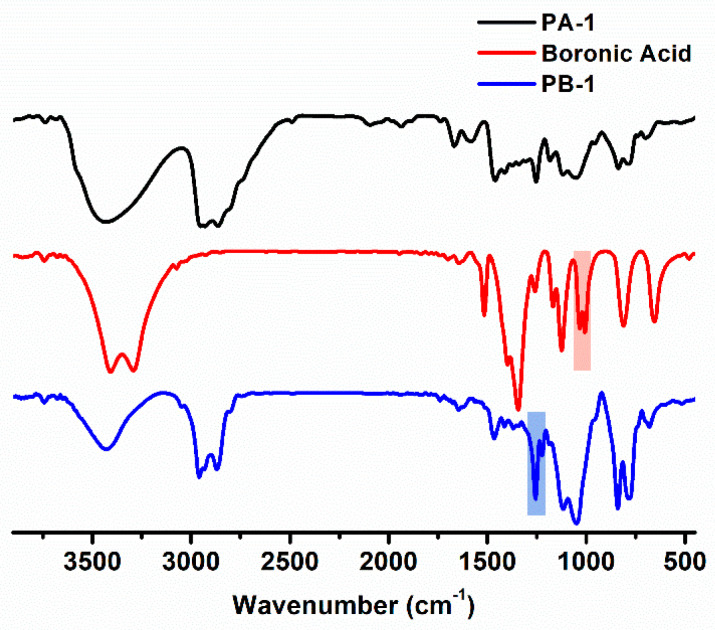
FT-IR spectra of **PA-1**, benzene-1,4-diboronic acid and **PB-1**. (The red box represents the absorption peaks corresponding to B-O stretching vibration and O-H in-plane bending vibration, and the blue box represents the asymmetric vibrational peaks of the B-O bonds).

The formation of boronic ester bonds was further analyzed using FT-IR spectra (Figure 2). For material **PB-1**, the absorption peaks corresponding to B-O stretching vibration and O-H in-plane bending vibration of benzene-1,4-diboronic acid around 1000 cm^−1^ disappeared, while a new absorption peak emerged at 1225 cm^−1^, attributed to the asymmetric vibrational peaks of the B-O bonds.

### 3.2. Thermal and Optical Property of Boronic Ester Cross-Linked Materials

**Figure 3:**
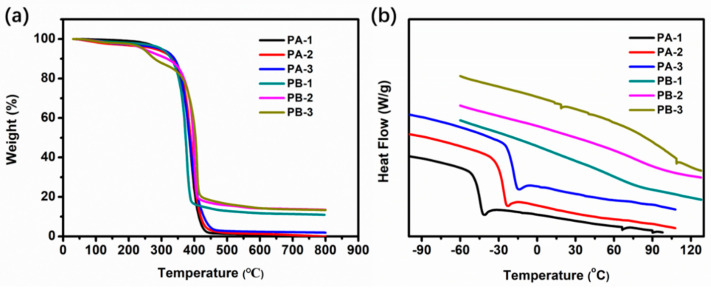
TGA (**a**) and DSC (**b**) curves of **PA-1**, **PA-2**, **PA-3**, **PB-1**, **PB-2** and **PB-3**.

The thermal stability of both poly(ß-hydroxyl amine)s and their materials was investigated using thermogravimetric analysis (TGA). As shown in Figure 3a, the 5 wt% thermal weight loss temperatures exceeded 240 °C, demonstrating good thermal stability. In the temperature range from 450 °C to 800 °C, the residual weight percentage values in Table 1 remained constant for both poly(ß-hydroxyl amine)s and their materials. The values were nearly 0% for poly(ß-hydroxyl amine)s, while they reached around 12% for their boronic ester cross-linked materials. It was presumed that the poly(ß-hydroxyl amine)s would easily degrade into volatile compounds at high temperatures, while the phenylboronic ester in their materials would likely form non-volatile phenyl boroxine structures, leading to higher residual weight percentage values.

Additionally, differential scanning calorimetry (DSC) measurements were utilized to characterize the thermal phase transition of poly(ß-hydroxyl amine)s and their materials. As shown in Figure 3b, apparent glass transitions were observed for all the polymers, with glass transition temperature (T_g_) values of −46 °C, −28 °C, and −20 °C for polymers **PA-1**, **PA-2**, and **PA-3**, respectively. The increasing trend in T_g_ values was attributed to the higher content of hydroxyl groups, which increased hydrogen-bonding interactions and consequently constrained the segmental motion of polymer chains. Upon the addition of benzene-1,4-diboronic acid, the T_g_ values of the resulting materials substantially increased to 63 °C, 77 °C, and 95 °C, respectively, indicating that the cross-linking networks significantly restricted the movement of polymer segments.

**Figure 4:**
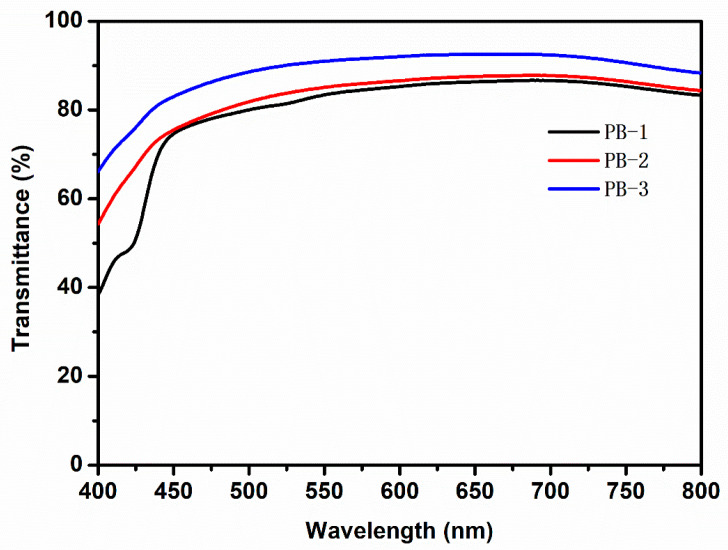
Visual transmittance of **PB-1**, **PB-2,** and **PB-3**.

The transmittance of three materials was tested, as shown in Figure 4. The visible light transmittance of the three plastics obtained was all above 80%, indicating good visible light transmittance.

### 3.3. Mechanical Property of Boronic Ester Cross-Linked Materials

**Figure 5:**
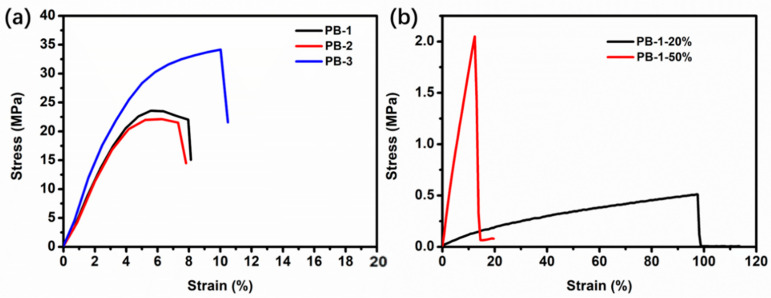
Stress–Strain curves of **PB-1**, **PB-2**, **PB-3** (**a**), **PB-1-20%** and **PB-1-50%** (**b**).

The mechanical characteristics of the boronic ester cross-linked polymers were assessed through static tensile stress–strain measurements. As shown in Figure 5a, with a fixed molar ratio of nitrogen atoms to boron atoms at 1:1, both **PB-1** and **PB-2** exhibited stress at a break of approximately 22 MPa, while **PB-3** displayed notably higher stress at a break of 34 MPa, surpassing other reported dynamic siloxane-based materials. All three materials exhibited an elongation at a break of less than 10%. The high Young’s modulus values were 651 MPa, 628 MPa, and 734 MPa for materials **PB-1**, **PB-2**, and **PB-3**, respectively. Notably, **PB-3** demonstrated significantly higher tensile strength and elongation at break compared to **PB-1** and **PB-2**, potentially due to the presence of two hydroxyl groups from 3-amino-1,2-propanediol monomers, which could facilitate the formation of additional hydrogen bonds between polymer molecular chains, thereby enhancing intermolecular forces and tensile strength. Furthermore, the supramolecular interaction of hydrogen bonds could undergo reversible fracture reconstruction during the stretching process, extending its elongation at break to a certain extent [26]. These findings provide valuable insights into the future development of dynamic polysiloxane materials with adjustable mechanical properties.

The impact of cross-linking density on the mechanical properties of the resulting PB materials was further explored. For poly(ß-hydroxyl amine) **PA-1**, two boronic ester cross-linked materials, **PB-1-20%,** and **PB-1-50%**, were prepared with fixed molar ratios of nitrogen atoms to boron atoms at 1:0.2 and 1:0.5, respectively. As depicted in Figure 5b, with increasing molar content of phenylboronic acid groups, the tensile strength increased from 0.51 MPa to 2 MPa, while the elongation at break decreased from 98% to 13%. Notably, the **PB-1** material with a molar ratio of nitrogen atoms to boron atoms fixed at 1:1 exhibited optimal mechanical properties, with a tensile strength of 23.6 MPa and an elongation of 7.9%. This demonstrates the potential for adjusting the mechanical properties of these materials by simply controlling the amount of benzene-1,4-diboronic acid, in addition to modifying the chemical structures of poly(ß-hydroxyl amine)s.

### 3.4. Dynamic Properties of Boronic Ester Cross-Linked Materials

As a dynamic covalent bond, boronic esters undergo reversible hydrolysis, alcoholysis, and transesterification. With sample **PB-1** as an example, we further investigated the dynamic properties of these boronic esters cross-linked materials, including solvent-assisted healing, hot-pressing reprocessing, and solution processing.

**Figure 6:**
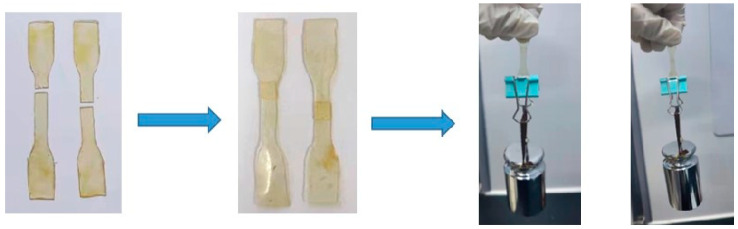
Healing of **PB-1** assisted by ethanol at room temperature.

The intrinsic dynamic nature of boronic esters confers healing capabilities to the material. As depicted in Figure 6, a dumbbell-shaped **PB-1** sample was cut into two pieces, subsequently wetted with a minute quantity of ethanol, and partially stacked together. The samples were rejoined, incubated at room temperature for 24 h, and successfully supported a 500 g load without fracturing, thereby exemplifying the effective solvent-assisted healing attributes of **PB-1**. At the surface of **PB-1**, external ethanol molecules exchanged with its boronic esters, resulting in the generation of boronic acid groups and alcohols within the polymeric network. Upon contact, the boronic acids and hydroxyl groups reacted to form new boronic ester cross-linkages at the interface, realizing the macroscopic healing of the plastic material.

**Figure 7:**
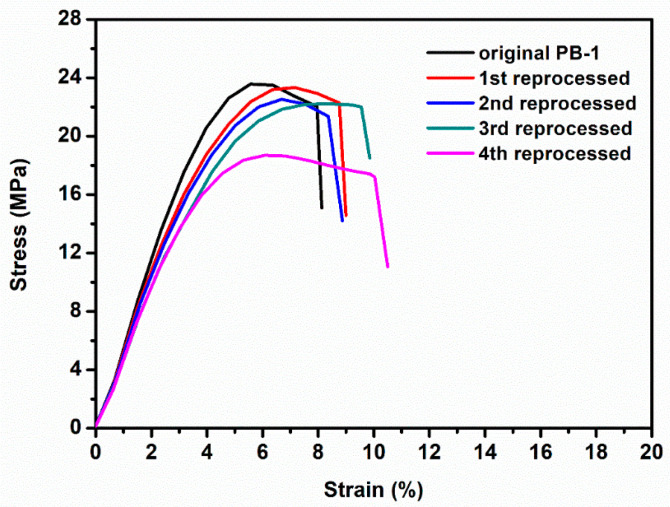
Stress–strain curves of original and reprocessed **PB-1** after hot-pressing four times.

Furthermore, the reprocessing potential through hot-pressing serves as an additional indicator of the dynamic reversibility of the polymer materials. The **PB-1** sample was cut into pieces and then subjected to hot-pressing at 150 °C and 12 MPa for 30 min. Subsequently, following cooling and demolding, the sample **PB-1** was cut into a dumbbell-shaped sample for tensile testing (Figure 7). After undergoing three successive hot-pressing cycles, the mechanical properties of the sample exhibited minimal alteration, with the tensile strength and elongation at break essentially restored. Even after the fourth hot-pressing, the tensile strength could still recuperate to 80% of the original sample, thereby demonstrating the exceptional reprocessing capability of **PB-1**. During the hot-pressing process, both heat and pressure augmented the segmental motion and the exchange of boronic esters, rendering the dynamic covalent network of **PB-1** malleable.

**Figure 8:**
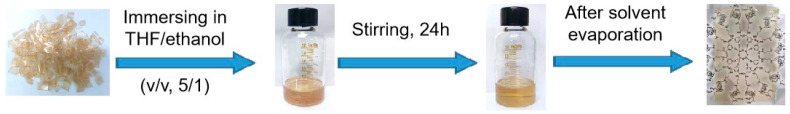
Schematic diagram of the dissolution and regeneration of **PB-1**.

**Figure 9:**
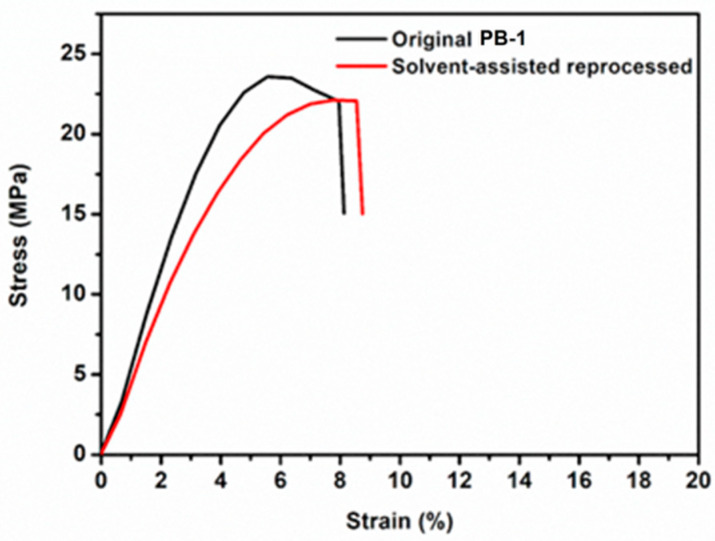
The stress–strain curves of original **PB-1** and reprocessed sample after dissolution and solvent evaporation.

In addition to hot-pressing, the chemical recycling of these boronic ester cross-linked materials was further explored. Sample **PB-1** was immersed in a mixed solution of THF and ethanol (*v*/*v*, 5/1) (Figure 8). After 24 h, the sample completely dissolved due to the alcoholysis of boronic ester bonds induced by ethanol, leading to the disruption of the cross-linking network of **PB-1**. Following solvent evaporation and subsequent drying at 60 °C for 24 h, the boronic ester cross-linked material was regenerated and subjected to stress–strain testing. As depicted in Figure 9, the ultimate stress of the new sample restored 94% of the original value. Given its mild temperature and pressure requirements, chemical recycling presents an appealing alternative to hot-pressing for reprocessing these boronic ester cross-linked materials.

### 3.5. Shape Reconfigurability of Boronic Ester Cross-Linked Materials

The three-dimensional (3D) configurations of polymers play a pivotal role in enabling their practical utility across diverse applications, including soft robotics, deployable devices, and aerospace materials. The dynamic covalent nature of these boronic ester cross-linked materials allows for the fabrication of 3D structures or topologies from flat sheet samples, which are challenging to achieve using traditional molding techniques. Leveraging solvent-induced bond exchange reactions and elastic-plastic transitions, the 3D shape programming of flat sample **PB-1** was demonstrated.

**Figure 10:**
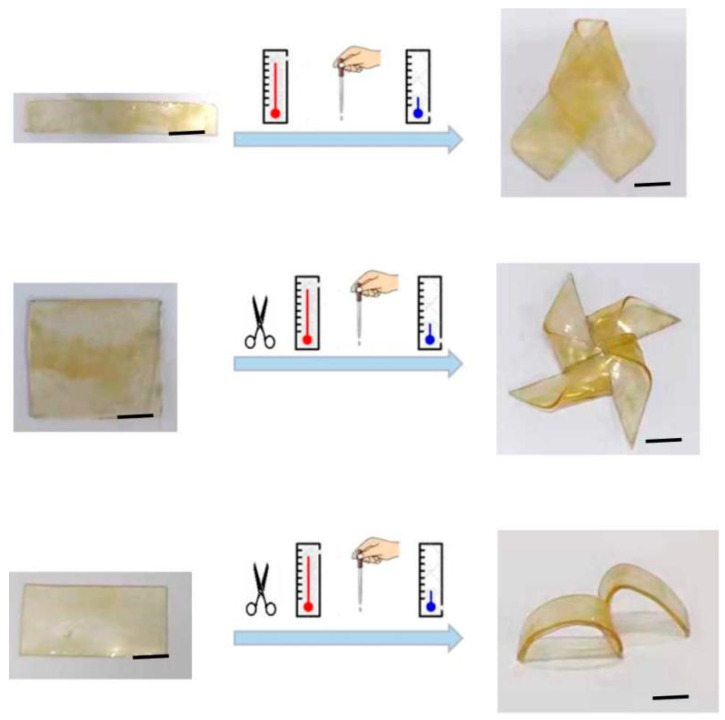
Schematic diagram of the ethanol-induced shape reconfiguration process (Scale bar, 500 μm).

As illustrated in Figure 10, the flat sheet of sample **PB-1** was heated to 80 °C (above T_g_) and bent into the desired 3D shape under external force. Subsequently, it was cooled to room temperature (below T_g_) to preserve the pre-strained shape, which could be automatically restored to its original flat shape upon heating above T_g_. To permanently fix this 3D shape, a small quantity of ethanol was applied to the inner and outer surfaces of the bent part. The solvent ethanol serves to fasten the exchange reaction rate of boronic ester bonds. Thanks to the solvent-induced exchanging of boronic esters, sample **PB-1** underwent network rearrangement and plastic deformation, thereby releasing the internal forces and locking the 3D shape upon the natural evaporation of ethanol. Notably, **PB-1** retained its new shape even upon subsequent heating. Hot-pressing under conditions of 150 °C and 12 MPa could transform the 3D shape into the original sheet shape. Furthermore, the sheet-like sample **PB-1** could be manipulated into various intricate 3D shapes using scissors and ethanol solvent in combination with a hair dryer. Leveraging the dynamic nature of boronic ester cross-linked materials, we offer a novel approach for achieving 3D structures or topologies, which are challenging to attain using traditional molding methods.

## 4. Conclusions

In conclusion, poly(ß-hydroxyl amine)s with abundant hydroxyl groups serve as a promising platform for constructing dynamic covalent materials, facilitated by the facile formation of boronic esters with phenylboronic acid. The introduction of nitrogen atoms within the polymeric chains further enhances the stability of boronic ester bonds due to the formation of B-N dative bonds. By varying the chemical structures of poly(ß-hydroxyl amine)s and the content of cross-linker, these materials exhibit adjusted thermal and mechanical properties, with a maximum T_g_ of 95 °C and a maximum tensile strength of up to 34.2 MPa. The intrinsic dynamic nature of boronic esters confers solvent-assisted healing, hot-pressing reprocessing, and chemical recycling capabilities to these materials. Additionally, the dynamic covalent nature of these materials allows for the fabrication of 3D structures from flat sheet samples, offering a novel approach to achieving intricate 3D shapes. These findings highlight the potential of boronic ester cross-linked polymers for various practical applications, including flexible electronic and biomedical materials.

## Figures and Tables

**Table 1:** Thermal properties of samples.

Sample	PA-1	PA-2	PA-3	PB-1	PB-2	PB-3
T_d,5%_ (°C)	297	281	297	301	245	250
T_g_ (°C)	−46	−28	−20	63	77	95
Residual weight (%)	0	0	2	11	13.5	13.4

## Data Availability

The original contributions presented in this study are included in the article. Further inquiries can be directed to the corresponding author.
